# Management of immune-related myocarditis, myositis and myasthenia gravis (MMM) overlap syndrome: a single institution case series and literature review

**DOI:** 10.3389/fimmu.2025.1597259

**Published:** 2025-05-08

**Authors:** Alberto Sánchez-Camacho, Alberto Torres-Zurita, Laura Gallego-López, Rocío Hernández-Pacheco, Silvia Silva-Romeiro, María del Carmen Álamo de la Gala, Enrique Peral-Gutiérrez de Ceballos, Luis de la Cruz-Merino

**Affiliations:** ^1^ Department of Medical Oncology, Hospital Universitario Virgen Macarena, Seville, Spain; ^2^ Autoimmune Disease Unit, Department of Internal Medicine, Hospital Universitario Virgen Macarena, Seville, Spain; ^3^ Institute of Biomedicine of Seville (IBiS), Hospital Universitario Virgen Macarena, CSIC, University of Seville, Seville, Spain; ^4^ Department of Medicine, University of Seville, Seville, Spain

**Keywords:** myositis, myocarditis, myasthenia gravis, immune-related adverse events, overlap syndrome, immune checkpoint inhibitors, MMM overlap syndrome

## Abstract

**Background:**

Immune checkpoint inhibitors (ICIs) have revolutionized the treatment of various malignancies, particularly melanoma. However, immune-related adverse events (irAEs) pose significant challenges, particularly in cases of severe toxicity syndromes. One such life-threatening irAE is the myocarditis, myositis, and myasthenia gravis (MMM) overlap syndrome, which occurs in less than 1% of patients but has in-hospital mortality rates ranging from 40 to 60%. Due to its rarity and complexity, early recognition and a multidisciplinary approach are critical to improving outcomes.

**Methods:**

We present a single-institution case series of four patients diagnosed with MMM overlap syndrome following ICI therapy. Clinical presentation, laboratory findings, imaging, and electrophysiological tests were analyzed to confirm the diagnosis. Therapeutic interventions-including corticosteroids, intravenous immunoglobulins (IVIG), plasma exchange (PLEX), tocilizumab, and rituximab- were evaluated in terms of efficacy and clinical outcomes.

**Results:**

The onset of MMM syndrome varied from 2 to 4 weeks after initiating ICI therapy. Patients presented with rapidly progressive symptoms, including ptosis, bulbar dysfunction, respiratory distress, myopathy, and cardiac conduction abnormalities. Immunosuppressive therapy with high-dose corticosteroids was initiated in all cases. Additional immunomodulatory treatments (IVIG, tocilizumab, PLEX, and rituximab) were administered based on clinical deterioration and autoimmune profile. Two patients achieved complete recovery, one remains on maintenance immunosuppression, and one died due to respiratory failure despite aggressive treatment.

**Conclusion:**

MMM overlap syndrome is a severe and often fatal irAE associated with ICI therapy. Early identification, aggressive immunosuppressive treatment, and individualized therapeutic strategies are essential to optimize patient outcomes. Further research is needed to refine diagnostic criteria, identify predictive biomarkers, and establish standardized treatment protocols.

## 1 Introduction

Immune checkpoint inhibitors (ICIs) have become a cornerstone of cancer therapy over the last two decades. The introduction of ICIs targeting key immune checkpoints such as cytotoxic T-lymphocyte-associated antigen 4 (CTLA-4), programmed cell death protein 1 (PD-1) on T cells, its ligand on tumor cells (PD-L1), or recently the lymphocyte activation gene-3 (LAG-3) have become integral components in the treatment of malignant melanoma.

These agents enhance the immune response against tumors leading to substantial improvements in overall survival rates among patients with malignant melanoma (1–3).

The efficacy of ICIs in melanoma was first highlighted with the approval of ipilimumab, an anti-CTLA4 antibody which marked a milestone in cancer immunotherapy. This study demonstrated that ipilimumab significantly improved overall survival in patients with advanced melanoma compared to a control group receiving a vaccine (4).

Subsequent studies have shown that PD-1 inhibitors, such as nivolumab and pembrolizumab, also provide durable responses and improved survival outcomes in patients with localized and advanced melanoma (5–9). Moreover, the clinical success of ICIs has led to the approval of combination therapies targeting different immune checkpoints. These include nivolumab and ipilimumab (anti-PD-1 and anti-CTLA-4, respectively), as well as the combination of nivolumab with relatlimab-rmbw (anti-LAG-3) (10, 11).

While ICIs have shown remarkable clinical efficacy, their use across various malignancies is associated with a wide range of different, and sometimes life-threatening, immune-related adverse events (irAEs) (3, 12). The majority of these irAEs are due to an overstimulation of the immune system, resulting in inflammation of different organs. Consequently, concurrent multiple irAEs are commonly observed, with incidence rates of 10-20% for monotherapy and up to 30-40% for combination regimes of ICIs (13, 14).

The overlap syndrome of myocarditis, myositis and myasthenia gravis (MMM) is a rare complication observed in approximately 0.1-0.3% of patients undergoing ICI therapy. Despite its low incidence, it is an extremely severe condition, with in-hospital mortality rates ranging from 38% to 60% (15). This highlights the critical importance of early detection and aggressive treatment to mitigate its potentially fatal outcomes.

The optimal management of this syndrome remains uncertain and relies on a combination of immunosuppressive therapies, including corticosteroids (CS), intravenous immunoglobulins (IVIG), plasma exchange (PLEX), and monoclonal antibodies, such as rituximab and tocilizumab (16–18). However, there is a lack of evidence regarding the most appropriate treatment regimen and dosing strategy tailored to the patient’s immunological profile. Therefore, we present a series of case reports involving patients with metastatic melanoma and other malignancies who developed MMM syndrome following ICI-based therapy. Our analysis focuses on treatment protocols and the immunological status of the patients, and it is supported by a comprehensive literature review of this rare condition.

## 2 Methods

We conducted a retrospective case series study at Hospital Universitario Virgen Macarena, analyzing patients diagnosed with ICI-induced myocarditis, myositis, and myasthenia gravis overlap syndrome between 2022 and 2024. Patients were identified through clinical records and hospitalization registries in the Department of Medical Oncology.

For each patient, we collected demographic characteristics, clinical and pathologic data related to malignancy diagnosis, previous treatments and past medical history. A comprehensive evaluation of each patient was conducted, including clinical symptoms, time of onset, ICI regimen, laboratory findings, cardiovascular assessments, imaging studies, electromyography (EMG), and autoantibody profiles. We focused on the immunosuppressive regimens administered in each case and their associated clinical outcomes. Treatment response was evaluated based on symptom resolution, biomarker normalization, and functional recovery. Mortality and long-term sequelae were also assessed.

## 3 Case report

### 3.1 Case 1

A 75-year-old male diagnosed in October 2022 with lentigo maligna melanoma on the left arm, stage IIIB (pT1aN1cM0), harboring a BRAF V600E mutation. Due to the lack of national funding in the adjuvant setting, a close clinical and radiological follow-up was decided. In March 2023, during follow-up after excision of a pigmented lesion in the cervical region, histopathological examination confirmed a melanoma metastasis. FDG-PET scan identified two brain lesions: a 25 x 20 mm left temporal lesion with perilesional edema but no hydrocephalus or midline shift, and a 9 mm right temporoparietal lesion without associated edema. Given the patient’s asymptomatic status and no immediate need for CS, a multidisciplinary oncology committee recommended the combination of nivolumab (1 mg/kg) plus ipilimumab (3 mg/kg) every 3 weeks.

Fifteen days after the first treatment cycle, the patient presented to the emergency department with acute neurological deterioration, including blurred vision, bilateral diplopia, instability, and syncope. An urgent cranial computed tomography (CT) scan revealed increased perilesional edema, without new lesions, prompting the initiation of high-dose intravenous CS (dexamethasone 4 mg every 8 hours).

During hospitalization, the patient developed acute chest pain and dyspnea. Electrocardiography (ECG) demonstrated complete atrioventricular block with a wide QRS escape rhythm resembling a right bundle branch block at 25 beats per minute, requiring initiation of an aleudrin infusion, transfer to the Intensive Care Unit (ICU), and permanent pacemaker implantation. Laboratory findings revealed grade 3 transaminitis (7 times the upper limit of normal [ULN]), elevated creatine kinase (CK) levels of 2187 U/L (11x ULN), and markedly elevated cardiac troponin-I (cTnI) levels of 1028 ng/L (64x ULN). Coronary angiography excluded an ischemic etiology, and echocardiography showed preserved left ventricular ejection function (LVEF) with mild mitral and tricuspid insufficiency. Neurological examination revealed significant psychomotor slowing, mixed transcortical aphasia, bilateral ptosis, ophthalmoplegia, and fatigability of the oculo-palpebral muscles.

Given the high suspicion of immune-related MMM syndrome, high-dose intravenous methylprednisolone (1 g/day for 5 days) was initiated, along with pyridostigmine (60 mg/8h), and immunosuppressive therapy with IVIG (2 g/kg over 4 days).

Over the following 72 hours, the patient’s condition deteriorated progressively, developing worsening cervical weakness, dysphagia, bilateral ptosis, rhinolalia, and respiratory distress, ultimately requiring non-invasive mechanical ventilation. Autoimmune workup in both peripheral blood and cerebrospinal fluid (CSF) was largely negative, with negative anti-acetylcholine receptor (AChR) antibody, except for a weakly positive autoantibody against striated muscle (anti-titin) in the blood. Electroencephalography showed generalized slowing with low-amplitude activity and bursts of generalized delta activity, consistent with mild-to-moderate encephalopathy, and EMG revealed mild myopathic changes without evidence of neuromuscular transmission disorder. Given the persistent refractory symptoms, a single dose of intravenous tocilizumab (8 mg/kg) was administered.

Despite immunosuppressive therapy, respiratory failure progressed, necessitating orotracheal intubation, invasive mechanical ventilation, and vasopressor support. Given the poor response, PLEX was initiated. On the sixth day in the ICU, after four sessions of PLEX, clinical and biochemical improvements were noted, making extubation possible. However, 48 hours later, the patient experienced respiratory depression and worsening immune-related Myasthenia Gravis (irMG) symptoms. Given the lack of further viable therapeutic options, a shared decision was made to withdraw life-sustaining treatment, and the patient passed away 14 days after admission.

### 3.2 Case 2

A 42-year-old woman, with no known drug allergies or significant medical history, was diagnosed with malignant melanoma, stage IIIB (pT3aN2aM0). She was referred to medical oncology in October 2024. After therapeutic evaluations, she was enrolled in a clinical trial, initiating adjuvant treatment with pembrolizumab (200 mg every 3 weeks), with the first cycle starting on October 24, 2024. During a follow-up visit on November 21, 2024, laboratory tests revealed grade 1 hypertransaminasemia. The patient reported mild generalized weakness and bilateral ptosis. As a result, further laboratory investigations were performed, including CK levels of 2021 U/L (10.3x ULN) and cTnI of 608 ng/L (38x ULN).

Given the suspicion of MMM overlap syndrome, the patient was admitted to the medical oncology ward for further evaluation and management. Upon admission, due to the severity of the suspected clinical presentation, treatment with intravenous methylprednisolone (500 mg/day for 3 days) was initiated, followed by a gradual taper to a dose of 1 mg/kg/day, alongside pyridostigmine (30 mg/8h). The patient showed significant clinical improvement, with progressive resolution of generalized weakness, predominantly affecting the ocular muscles, and normalization of muscle injury markers (both CK and transaminase levels). However, cTnI levels remained persistently elevated.

To further characterize the syndrome, an autoimmunity panel was performed, which was negative for anti-AChR and anti-titin antibodies. Additionally, cardiac magnetic resonance imaging (MRI) revealed interstitial edema in the apical segments, suggestive of myocarditis. EMG demonstrated acute myopathic changes consistent with myositis, while a repetitive stimulation study showed no significant findings, likely due to the ongoing administration of pyridostigmine. Considering the persistent elevation of cTnI levels, a cycle of IVIG was administered (2 g/kg over 3 days). Following clinical stabilization, the patient was discharged on November 29, 2024, under close monitorization.

At subsequent follow-up visits, the patient exhibited persistently elevated cTnI levels, stabilizing at approximately 300 ng/L. After a multidisciplinary discussion with the Internal Medicine Department (Autoimmune Disease Unit), it was decided to add intravenous tocilizumab (at a dose of 8 mg/kg every 2 weeks), receiving two doses. Joint follow-up by both specialties was arranged. Subsequently, the patient was transitioned to subcutaneous tocilizumab (162 mg weekly), with acceptable tolerance. This treatment regimen has been maintained to date, leading to the normalization of cTnI levels. Concurrently, the oral CS therapy was progressively tapered and with complete discontinuation in February 2025, after 3 months of treatment. To date, the patient remains asymptomatic, with no recurrence of irAEs, and immunotherapy has been permanently discontinued.

### 3.3 Case 3

A 59-year-old woman with a medical history of Hashimoto’s thyroiditis, managed with levothyroxine, presented with disabling lower back pain, unintentional weight loss, and anemia. Following a diagnostic work-up, she was diagnosed with a metastatic clear cell renal cell carcinoma (ccRCC). The disease was categorized as poor risk according to the International Metastatic RCC Database Consortium (IMDC) risk criteria. As the patient was in good clinical condition, first line treatment with nivolumab (3 mg/kg) plus ipilimumab (1 mg/kg) every 3 weeks was initiated in October 2024.

Two weeks after the second cycle, the patient presented with bilateral ptosis, diplopia, dysphagia, dysphonia, and cervical muscle weakness. Neurological examination detected impaired lateral eye movement, bilateral fatigable ptosis, vocal fatigability after 30 seconds and decreased strength in the upper limbs.

Laboratory findings revealed markedly elevated cTnI levels of 614 ng/L (38x ULN) and CK levels of 4157 U/L (20x ULN). Given the suspicion of MMM overlap syndrome, initial treatment with intravenous methylprednisolone (2 mg/kg/day) and oral pyridostigmine (60 mg/6 hours) was initiated in the emergency department.

ECG showed sinus rhythm with ST-segment depression in leads II, V5 and V6, with multiple isolated supraventricular extrasystoles. Echocardiography demonstrated a non-dilated and preserved LVEF, while cardiac MRI revealed myocarditis without systolic dysfunction.

After 6 days of hospitalization without improvement in irMG symptoms, PLEX was performed.

However, on day 20, the patient experienced worsening symptoms of both irMG and myositis with a further elevation of cTnI levels. Autoimmune serology testing was negative for anti-AChR and anti-muscle specific tyrosine kinase (MuSK) antibodies, but positive for anti-titin antibodies in both serum and CSF. Immunosuppressive therapy targeting lymphocyte depletion was initiated using both IVIG (2 g/kg over 4 days) and rituximab (375 mg/m2 weekly for 4 weeks), leading to a rapid clinical improvement after the first dose.

The patient was finally discharged on day 25 with oral CS and pyridostigmine. Four months post-hospitalization, the patient achieved full recovery from myositis and showed partial improvement in irMG symptoms, with minimal residual bilateral diplopia. CS therapy was tapered to complete discontinuation, immunotherapy was permanently discontinued, and the malignancy remains in partial response.

### 3.4 Case 4

A 70-year-old male with stage IV gastric adenocarcinoma with supra and infra-diaphragmatic lymphatic involvement and microsatellite instability-high status initiated first-line pembrolizumab (200mg every 3 weeks) in May 2023. After the first cycle, he developed grade 1 fatigue and polymyalgia, which worsened following the second cycle, prompting an emergency department visit due to generalized weakness, particularly affecting cervical muscles. Initial evaluation was unremarkable except for cervical extension weakness. Laboratory workup revealed grade 3 transaminitis. He was admitted to the medical oncology ward and started on intravenous methylprednisolone (1 mg/kg), resulting in partial improvement of symptoms and a reduction of transaminitis levels to grade 2.

During hospitalization, new symptoms emerged, including bilateral ptosis, hypoxemic respiratory insufficiency requiring oxygen supplementation, mild dysphagia, and dysphonia, suggestive of irMG with bulbar involvement. Further workup revealed elevated CK levels of 485 U/L (3x ULN) and a markedly increased cardiac troponin T (cTnT) level of 1430 ng/L (89x ULN), while autoimmune serologies were negative. ECG showed a new first-degree atrioventricular block. EMG indicated a proximal inflammatory myopathic process without neuromuscular transmission disorder, and echocardiography showed preserved LVEF. Cardiac MRI revealed mild concentric left ventricular hypertrophy and subtle late gadolinium enhancement in the basal-mid lateral wall, consistent with an immune-related myocarditis-like process. These findings confirmed a severe immune-related MMM overlap syndrome induced by pembrolizumab.

Due to worsening of bulbar myasthenic signs and respiratory compromise, immunosuppressive therapy was intensified with high-dose methylprednisolone (1 g/day) and a single dose of intravenous tocilizumab (8 mg/kg). The patient was transferred to the ICU for close cardiac monitoring and respiratory support, receiving three additional pulses of methylprednisolone. Within 72 hours, he exhibited ECG normalization, reduced oxygen requirements and improvement in CK (from 485 U/L to 277 U/L) and cTnT (from 1430 ng/L to 828 ng/L) levels. Despite these improvements, irMG symptoms persisted, including cervical weakness, gait instability, ptosis, and dysphonia, with fluctuating severity. cTnT levels continued to rise, whereas cTnI levels remained within the normal range, suggesting that both cTnT and transaminases elevation likely resulted from muscle regeneration rather than from ongoing myocardial damage or liver inflammation. Given refractory irMG symptoms and lack of clinical improvement, IVIG (4 g/kg over 5 days) was administered.

Over the subsequent 10 days, gradual improvement was noted, allowing for the complete withdrawal of supplemental oxygen, tapering of CS therapy, and stabilization of laboratory parameters, including normalization of transaminases, CK, and reduction of cTnT levels to below 500 ng/L. After 30 days of hospitalization, the patient was clinically stable and discharged.

## 4 Discussion

### 4.1 Immune-related adverse events in immunotherapy: general and specific considerations in melanoma

Immune-related adverse events represent a wide spectrum of toxicities associated with ICIs based therapies. These events are defined as side effects resulting from non-specific activation of the immune system against both tumor cells and normal tissues. irAEs can affect virtually any organ system are classified based on severity and timing using standardized criteria such as the Common Terminology Criteria for Adverse Events (CTCAE). Severity ranges from mild (grade 1) to life-threatening (grade 4), guiding treatment decisions accordingly (13, 19).

The prevalence of irAEs varies depending on the specific ICI used and cancer type, with studies estimating an overall incidence of 54-76% for all-grade events and approximately 21% for severe (grade 3/4) irAEs (20, 21). The most common manifestations are skin-related toxicities (especially pruritus or rash), followed by gastrointestinal effects (such as diarrhea and colitis), endocrine dysfunction (including thyroid disorders and adrenal insufficiency), musculoskeletal symptoms (such as arthralgia or myalgia) and hepatic abnormalities (hepatitis and transaminitis). Although rare, serious irAEs such as pneumonitis, myocarditis, neurotoxicity, myositis, nephritis, and hematological toxicity warrant special attention due to their potential seriousness (22).

Although the overall incidence of irAEs is comparable across different cancer types, the specific distribution of toxicities varies depending on both the type of ICI used and the malignancy being treated. For instance, while PD-1 inhibitors demonstrate similar irAE rates in melanoma and lung cancer, melanoma patients exhibit a higher prevalence of dermatologic (e.g., vitiligo) or gastrointestinal irAEs, whereas lung cancer and renal cell carcinoma patients are more prone to pneumonitis. This suggests that both ICI class and tumor-specific immune interactions contribute to the variability in irAE profiles (23).

PD-1 and PD-L1 inhibitors generally exhibit a more favorable safety profile compared to CTLA-4 and LAG-3 inhibitors, with systematic reviews showing grade 3/4 irAEs occurring in 31% of patients receiving CTLA-4 inhibitors versus 10% with PD-1/PD-L1 inhibitors. Specifically, colitis (Odds ratio [OR] 8.7, 95% Confidence interval [CI] 5.8–12.9), hypophysitis (OR 6.5, 95% CI 3.0–14.3) and rash (OR 2.0, 95% CI 1.8–2.3) were more common with CTLA-4 inhibitors, whereas pneumonitis (OR 6.4, 95% CI 3.2–12.7), hypothyroidism (OR 4.3, 95% CI 2.9–6.3), arthralgia (OR 3.5, 95% CI 2.6–4.8) and vitiligo (OR 3.5, 95% CI 2.3–5.3) were more common with PD-1 inhibitors (23, 24).

Combination immunotherapy regimens have enhanced the antitumor efficacy across a broad range of malignancies. However, these combinations are associated with a higher incidence of irAEs compared to monotherapy. The combination of CTLA-4 inhibitors with PD-1/PD-L1 inhibitors has been associated with grade 3/4 irAEs in approximately 60% of patients, while the combination of LAG-3 inhibitors plus PD-1 inhibitors has demonstrated grade 3/4 irAES rates of 40%. In contrast, monotherapy with ICIs results in grade 3/4 irAE rates of 20-30% (10, 11).

Several factors have been identified as predisposing to the development of irAEs, including a history of autoimmune disease, treatment with CTLA-4 inhibitors, and impaired renal function (grade ≥3) (25). Genetic predisposition, such as certain HLA alleles, also increases susceptibility to autoimmune events during ICI therapy (26). Specific biomarkers, such as baseline levels of certain cytokines (e.g. IL-17) or reduced baseline levels of others (e.g. IL-6) have been linked to an increased incidence of irAE (27). Additionally, gut microbiota composition may influence both ICI efficacy and toxicity. For example, a higher abundance of *Bacteroides* species has been correlated with a reduced risk of colitis in melanoma patients receiving ipilimumab, while *Faecalibacterium* has been associated with both improved outcomes and an increased risk of ICI-related colitis (28–30).

### 4.2 MMM overlap syndrome: manifestation and diagnosis

The MMM overlap syndrome secondary to ICIs is a rare toxicity with high morbidity and mortality that requires early and multidisciplinary therapeutic management due to its complexity. irMG, myositis, and immune-mediated myocarditis individually are infrequent side effects, with an incidence of less than 1%, but when they occur, it is not uncommon for them to present concurrently. It has been observed that up to 30-40% of irMG cases are associated with myositis, and up to 8-39.7% with myocarditis. The latter significantly worsens the prognosis, with reported mortality rates for MMM overlap syndrome ranging from 40-60%, according to published series (17, 18, 31–34).

The pathophysiology of MMM overlap syndrome remains unclear, although it is hypothesized that the molecular similarity between the myocyte, cardiomyocyte, and neuromuscular junction, combined with the dysregulation of ICIs affecting autoimmunity protective mechanisms and alterations in the PD-1/PD-L1 axis, could be the primary cause. Increased CD8+ T lymphocytes and a reduced number of CD4+ T cells have been observed in biopsies of immune-mediated myocarditis and myositis, with focal expression of PD-L1 in damaged cardiomyocytes, consistent with the direct mechanism of action of antibodies targeting PD-1/PD-L1 (33). Additionally, complement activation has been suggested to play a role in this toxicity, with C5b-9 deposits in muscle biopsies from an MMM case responding to eculizumab (a humanized monoclonal antibody that targets complement C5) (35), and pericapillary C4d deposits in immune-mediated myocarditis cases (36). Furthermore, a predisposition to developing this adverse event or irMG may exist when autoantibodies against the AChR or striated muscle are present prior to ICIs therapy, with antibody levels increasing due to immune system hyperactivation induced by ICIs (37–41). It does not appear that irMG cases are triggered by the coexistence of a thymoma (42).

Understanding the pathophysiology of MMM overlap syndrome is crucial for implementing targeted immunosuppressive treatment in cases mediated by complement or PD-1/PD-L1 axis disruption. Patients with positive autoantibodies prior to receiving ICIs may be predisposed to developing this adverse event and could benefit from preventive strategies or close monitoring (37–41).

MMM overlap syndrome typically occurs early in ICI therapy, with a median onset of 21 days after starting treatment, being more common in males and treatment with PD-1 inhibitors, with a median age of onset of approximately 70 years. The majority of reported cases were in patients diagnosed with melanoma. The most commonly reported signs and symptoms include dyspnea, diplopia, myalgias, muscle weakness, and dysphagia. Up to 40% and 11% may present arrhythmias or reduced LVEF, respectively (17, 18). Diagnosing irMG can be challenging, as myositis can present with symptoms that mimic myasthenia. Furthermore, up to a third of irMG patients lack detectable autoantibodies, while 60% may have normal electrophysiological studies (34). Considering the early onset of this adverse event, routine assessment of CK levels during the initial cycles of treatment may be warranted. **Table 1** provides a summary of the patient’s clinical presentation of MMM overlap syndrome, including symptom onset, details of ICI therapy, and relevant laboratory findings.

**Table 1:** Patient’s clinical presentation, date of first symptoms, ICI therapy and laboratory findings.

Case	Start Date of Symptoms	ICI Type	Laboratory Findings	irMG	Myositis	Myocarditis
Case 1	15 days post-ICI initiation	Nivolumab + Ipilimumab	- CK: 2187 U/L (11x ULN). - cTnI: 1028 ng/L (64x ULN). - Grade 3 transaminitis	- Bilateral ptosis. - Diplopia - Dysphagia. - Rhinolalia - Respiratory distress	- Cervical weakness. - Generalized muscle weakness	- Chest pain - Complete AV block with RBBB
Case 2	4 weeks post-ICI initiation	Pembrolizumab	- CK: 2021 U/L (10.3x ULN. - cTnI: 608 ng/L (38x ULN) - Grade 1 transaminitis	- Bilateral ptosis.	- Proximal muscle weakness.	- Interstitial edema on cardiac MRI
Case 3	5 weeks post-ICI initiation	Nivolumab + Ipilimumab	- CK: 4157 U/L (20x ULN). - cTnI: 614 ng/L (38x ULN)	- Bilateral ptosis. - Diplopia. - Vocal fatigability - Rhinolalia	- Cervical weakness. - Upper limb weakness	- Myocarditis on cardiac MRI
Case 4	6 weeks post-ICI initiation	Pembrolizumab	- CK: 483 U/L (3x ULN). - cTnT: 1430 ng/L (89x ULN) - Grade 3 transaminitis	- Bilateral ptosis. - Dysphonia. - Dysphagia	- Cervical weakness - Generalized weakness	- 1st-degree AV block. - Late gadolinium enhancement on MRI

AV, atrioventricular; CK, creatine kinase; cTnI, cardiac troponin I; cTnT, cardiac troponin T; ICI, immune checkpoint inhibitor; irMG, immune-related myasthenia gravis, MRI, magnetic resonance imaging; RBBB, right bundle branch block; ULN, upper limit of normal.

Given the frequent overlap, identifying one of the three toxicities separately warrants investigation of the other two. Therefore, for the diagnosis of each component, the following is recommended:

#### 4.2.1 Immune-mediated myocarditis

ICI-induced myocarditis is a life-threatening toxicity characterized by myocardial inflammation and conduction abnormalities. Diagnosis is often based on clinical symptoms (fatigue, dyspnea, chest pain, palpitations, syncope), analytical findings (elevated cTnI and pro-B-type Natriuretic Peptide [pro-BNP] levels), and through imaging techniques such as cardiac MRI. Periodic ECG are essential to detect conduction disorders, and echocardiography should be performed to monitor LVEF. The International Cardio-Oncology Society has published a consensus for diagnosis, establishing a significant elevation in troponins and either a cardiac MRI compatible with acute myocarditis, or alternatively, two of the following criteria: clinical compatibility, ventricular arrhythmia, new conduction disturbances, reduced LVEF, or the presence of another immune-mediated adverse event (myositis, myasthenia, or myopathy), excluding acute coronary syndrome or an infectious cause for myocarditis (43). The definitive diagnosis is obtained through endomyocardial biopsy, which typically shows infiltration by CD8+ and CD4+ T lymphocytes and CD68+ macrophages, with variable cardiomyocyte damage (18).

#### 4.2.2 irMG

Diagnosis relies on clinical presentation (with muscle weakness and fatigability on repetitive exertion that improves with rest), serological findings of autoantibodies against the neuromuscular junction (anti-AChR and anti-MuSK) or against striated muscle (anti-titin), and electrophysiological findings showing a decremental response in action potentials to repetitive stimulation. Severity of irMG symptoms should be categorized using the Myasthenia Gravis Foundation of America (MGFA) classification, as this has prognostic implications (31) and helps determine when to intensify immunosuppressive treatment (44).

#### 4.2.3 Myositis

Diagnosis is determined by clinical symptoms, with myalgias, proximal muscle weakness, and analytical evidence of elevated muscle enzymes (CK, aldolase, transaminases, and lactate dehydrogenase). Radiological findings on MRI of the affected muscle or EMG showing myopathy may also support the diagnosis. Confirmation is provided by muscle biopsy, which typically shows myonecrosis and infiltration by CD8+ T lymphocytes and macrophages (18).

#### 4.2.4 Differential diagnosis

Each of the three components should be considered separately in the differential diagnosis of MMM overlap syndrome,. Regarding musculoskeletal toxicity induced by ICIs, a differential diagnosis should include entities such as neuromyopathy, arthritis, fasciitis, tenosynovitis, rhabdomyolysis, polymyalgia rheumatica, and chondrocalcinosis (45). In terms of neuromuscular junction disorders, irMG patients present with more clinical impairment (MGFA class III-IV) compared to classic myasthenia (34), evolving rapidly into myasthenic crisis requiring respiratory support. A differential diagnosis should be made with Lambert-Eaton myasthenic syndrome (LEMS) associated with ICIs, due to clinical and presentation similarities. The main characteristics of LEMS is that in most cases, it is mediated by antibodies against voltage-gated calcium channels, with primary involvement of proximal muscles that improve with exercise and a very positive response to repetitive stimulation in EMG. Another neuropathic toxicity resembling irMG is immune-mediated Guillain-Barré syndrome (GBS), which typically presents with a clinical course similar to classic GBS (46). Most cases are acute inflammatory demyelinating polyneuropathies, athough less common variants such as Miller-Fisher syndrome have also been reported in association with ICIs (47). Typical GBS symptoms include progressive ascending weakness that does not improve with rest, possibly associated with paresthesia or sensory changes. The diagnosis does not rely on specific antibodies for GBS; however, albuminocytologic dissociation in CSF and neurophysiological studies suggest an underlying demyelinating process, although acute axonal processes have also been reported (48). Finally, the differential diagnosis of immune-mediated myocarditis is crucial because its symptoms may overlap with other cardiovascular pathologies. The most common cardiovascular adverse events induced by ICIs include heart failure (5.3%), atrial fibrillation (4.6%), acute myocardial infarction (2.0%), atrioventricular block (1.9%), myocarditis (1.2%), vasculitis (0.8%), pericarditis (0.4%), and Takotsubo cardiomyopathy (<0.3%) (49).

### 4.3 MMM overlap syndrome: management

ASCO and ESMO guidelines for irMG or myositis recommend early initiation of CS at a dose of prednisone 0.5 mg/kg/day for mild cases (G2 or MGFA I and II), along with pyridostigmine in irMG, and methylprednisolone 1–2 mg/kg/day for severe myositis cases (G3-4) or IVIG or PLEX in irMG with MGFA class III-V. For myocarditis, CS pulses with methylprednisolone at doses of 500 mg–1000 mg per day are recommended. In cases of refractoriness to any of the three entities, escalation to more potent immunosuppressive drugs, such as tocilizumab, is recommended, as was done in cases 1 and 2 (12, 50).

In the case of MMM, series with the largest number of cases (17, 18) describe this sequence of recommendations in the guidelines, where 98% of patients received CS at varying doses, 52% received IVIG, and 36% received PLEX, with mycophenolate being the most commonly used immunosuppressive therapy, in up to 18% of cases. Despite this, the hospital mortality rate for MMM syndrome remains between 40-60%. This highlights the importance of early identification and immunosuppressive treatment initiation, as well as multidisciplinary management due to its high diagnostic and therapeutic complexity. Additionally, it is crucial to tailor immunosuppressive therapy to each of the three toxicities, prioritizing the more severe or worsening condition. Similarly, identifying the triggering cause will help guide targeted immunosuppressive treatment, improving the prognosis.

Early immunosuppression was described by Weaver et al. (44), with 10 cases initially treated with methylprednisolone 2 mg/kg/day, and in those with MGFA class II or higher, IVIG 1–2 g/kg divided over five days was added from the outset, resulting in a mortality rate of 10%. This early immunomodulation by IVIG could prevent the potential initial worsening of irMG secondary to CS, recommending its use from the beginning alongside these (34). **Table 2** provides a comprehensive summary of the patient’s autoimmune profile and immunosuppressive therapy regimen used in our cohort of MMM overlap syndrome cases.

**Table 2:** Patient’s autoimmune profile and immunosuppressive therapy patterns.

Case	Autoantibody findings	Start Date	Therapy	Duration/Remarks
Case 1	Anti-titin + (blood)	Hospital admission	Dexamethasone (4mg/8h)	Continued
			Pyridostigmine (60 mg/8h)	Continued
		Day 3	High-dose Methylprednisolone (1g/day)	5 days, then tapered to 1 mg/kg/day
			IVIG (2 g/kg)	Over 4 days
		Day 6	Tocilizumab (8 mg/kg)	Single dose
		Day 7	PLEX	5 sessions
Case 2	None	Hospital admission	High-dose Methylprednisolone (500 mg/day)	3 days, then tapered to 1 mg/kg/day
			Pyridostigmine (30 mg/8h)	Continued
			IVIG (2 g/kg)	Over 3 days
		Day 8	Tocilizumab IV (8 mg/kg every 2 weeks)	2 doses
		After IV therapy	Tocilizumab SC (162 mg weekly)	2 doses
Case 3	Anti-titin +++ (blood and CSF)	Hospital admission	Methylprednisolone (2 mg/kg/day)	Continued
			Pyridostigmine (60 mg/6h)	Continued
		Day 6	PLEX	5 sessions
		Day 20	IVIG (2 g/kg)	Over 4 days
			Rituximab (375 mg/m² weekly)	4 weeks
Case 4	None	Hospital admission	Methylprednisolone (1 mg/kg/day)	Continued
		Day 3	High-dose Methylprednisolone (1 g/day)	4 days, then tapered to 1 mg/kg/day
			Tocilizumab (8 mg/kg)	Single dose
		Day 7	IVIG (4 g/kg)	Over 5 days

CSF, cerebrospinal fluid; IV, intravenous; IVIG, intravenous immunoglobulin; PLEX, plasma exchange.

Regarding identifying the triggering cause, preclinical models have suggested that the loss of one heterozygous allele in the CTLA4 gene may play a significant role in predisposition to developing immune-mediated myocarditis. Therefore, restoring this mechanism using abatacept, a CTLA4 immunoglobulin fusion protein, combined with faster-acting immunosuppressors such as ruxolitinib, a Janus kinase (JAK) inhibitor that inhibits T cell activation, reduced mortality from 60% to 3% in a series of 40 immune-mediated myocarditis cases (51). As previously mentioned, cases with positive autoantibodies before ICI use have been identified with increased levels during MMM, so if there is no improvement with the initial CS and IVIG strategy, one consideration is using immunosuppressors that deplete these autoantibodies, such as rituximab, which acts on CD20+ B lymphocytes, as we did in our case 3, or efgartigimod, approved for use in MG patients with anti-AChR positivity (52). In other MMM cases, complement has been identified as playing an important role in its development (35, 36), justifying the use of targeted drugs such as eculizumab or ravulizumab. PLEX does not appear to be an effective strategy for this toxicity, either due to its late use or in more severe patients, and it may decrease the levels of previously used immunosuppressors (31, 51).

This study has several limitations that should be acknowledged. First, it is a retrospective analysis with a small sample size, including only four patients, which limits the generalizability of the findings. Second, the patient cohort is heterogeneous, comprising individuals with varying tumor types who were treated at different time points with different ICIs. As a result, the diagnostic work-up and therapeutic approaches varied across cases, reflecting differences in clinical context and treatment availability at the time. These factors may introduce variability that affects the interpretation and comparability of outcomes.

## 5 Conclusion

Overlap syndrome of myocarditis, myositis and myasthenia gravis represents a rare but potentially life-threatening complication in patients receiving ICI therapy. Early recognition and aggressive treatment are crucial to reduce associated morbidity and mortality.

Effective management of MMM overlap relies on three key principles: (1) early identification and initiation of immunosuppressive therapy, including CS and IVIG, particularly in patients with MGFA class II or higher; (2) continuous assessment to identify the most severe evolving toxicity and prioritizing its management accordingly; and (3) identification of the potential triggering factors to guide targeted immunosuppressive strategies.

Given the complexity and high-risk nature of this syndrome, a multidisciplinary treatment approach and long-term follow-up are essential for optimizing patient outcomes and improving prognosis.
